# A community-level investigation following a yellow fever virus outbreak in South Omo Zone, South-West Ethiopia

**DOI:** 10.7717/peerj.6466

**Published:** 2019-02-20

**Authors:** Ranya Mulchandani, Fekadu Massebo, Fekadu Bocho, Claire L. Jeffries, Thomas Walker, Louisa A. Messenger

**Affiliations:** 1 Department of Disease Control, London School of Hygiene & Tropical Medicine, University of London, London, UK; 2 Department of Biology, Arba Minch University, Arba Minch, Ethiopia

**Keywords:** Yellow fever virus, Ethiopia, South Omo Zone, Outbreak, *Aedes simpsoni*, Knowledge attitudes and practices

## Abstract

**Background:**

Despite the availability of a highly effective vaccine, yellow fever virus (YFV) remains an important public health problem across Africa and South America due to its high case-fatality rate. This study investigated the historical epidemiology and contemporary entomological and social determinants of a YFV outbreak in South Omo Zone (SOZ), Ethiopia.

**Methods:**

A YFV outbreak occurred in SOZ, Ethiopia in 2012–2014. Historical epidemiological data were retrieved from the SOZ Health Department and analyzed. Entomological sampling was undertaken in 2017, including mosquito species identification and molecular screening for arboviruses to understand mosquito habitat distribution, and finally current knowledge, attitudes and preventative practices within the affected communities were assessed.

**Results:**

From October 2012 to March 2014, 165 suspected cases and 62 deaths were reported, principally in rural areas of South Ari region (83.6%). The majority of patients were 15–44 years old (75.8%) and most case deaths were males (76%). Between June and August 2017, 688 containers were sampled across 180 households to identify key breeding sites for *Aedes* mosquitoes. *Ensete ventricosum* (“false banana”) and clay pots outside the home were the most productive natural and artificial breeding sites, respectively. Entomological risk indices classified most sites as “high risk” for future outbreaks under current World Health Organization criteria. Adult mosquitoes in houses were identified as members of the *Aedes simpsoni* complex but no YFV or other arboviruses were detected by PCR. The majority of community members had heard of YFV, however few activities were undertaken to actively reduce mosquito breeding sites.

**Discussion:**

Study results highlight the potential role vector control could play in mitigating local disease transmission and emphasize the urgent need to strengthen disease surveillance systems and in-country laboratory capacity to facilitate more rapid responses to future YFV outbreaks.

## Introduction

Yellow fever virus (YFV) is a *flavivirus* transmitted primarily to humans and non-human primates through the bite of an infected female mosquito; *Aedes* spp. are responsible for transmission in Africa and the Americas, while *Haemagogus* spp. vectors are restricted to the latter region (Monath & Vasconcelos, 2015). Yellow fever (YF) causes a spectrum of clinical symptoms ranging in severity from mild illness with flu-like symptoms to severe disease including, fever, jaundice or haemorrhage, and death (Monath, 2001), for which there is no treatment. Despite the availability of a highly effective vaccine, which can confer lifelong immunity, YF continues to occur in epidemic situations, and it is estimated to result in 130,000 human cases and 78,000 deaths annually in Africa alone (Garske et al., 2014). In 2015–2016, urban outbreaks of YFV were declared in Angola and Democratic Republic of Congo, and a sylvatic outbreak has been ongoing in Brazil since late 2016 (Zhao et al., 2018; Kraemer et al., 2016; World Health Organization (WHO), 2017; Chippaux & Chippaux, 2018; Pan American Health Organization (PAHO), 2018).

Rapid urbanization, population migration, climatic changes, and increased travel have all been implicated in expanding the geographical range of YFV and bringing high densities of competent mosquito vectors close to considerable numbers of unvaccinated individuals (Hamrick et al., 2017). YF prevention and control requires strong laboratory and surveillance systems with rapid case detection and reporting, appropriate case management, efficient healthcare policies, including routine immunization of infants, vector control activities and both reactive and preventive vaccination campaigns (World Health Organization (WHO), 2003).

Ethiopia has experienced numerous YFV outbreaks since the 1940s. Between 1960 and 1962, the largest YFV outbreak ever recorded in Africa occurred along the River Omo, in South-West Ethiopia (Gamo Gofa, Jinka, and Kaffa regions) and resulted in approximately 200,000 human cases and 30,000 deaths (Serie et al., 1968). In 1966, YFV appeared in Arba Minch in South Ethiopia, in an area previously unaffected in the 1960 epidemic and therefore excluded from the mass vaccination campaign at the time. During this outbreak, 450 deaths were reported (2,200 human cases) and the outbreak was confirmed through serological testing (Ardoin, Rodhain & Hannoun, 1976). After almost a 50-year absence of reported human cases, YFV re-emerged in South Omo in 2012–2014. A reactive vaccination campaign commenced in June 2013 to control the outbreak, which reached approximately 550,000 people across the at-risk population. Most recently, following five confirmed cases of YFV in South-West Ethiopia in October 2018 (from a total of 38 suspected cases, including 11 deaths as of October 29, 2018), a reactive vaccination campaign was triggered targeting 31,365 people, with another planned to include 1.34 million people in nearby districts (World Health Organization (WHO), 2018). The outbreaks of 2012 and 2018 highlight the urgent need to scale-up long term prevention and control measures in this vulnerable region.

In Ethiopia, there is a considerable paucity of epidemiological and entomological data about YF. The population is highly susceptible to YFV due to a lack of recent exposure and large-scale vaccination and it is therefore important to understand its context to guide appropriate, prospective disease control interventions. The aim of this study, conducted in July and August 2017, was threefold, to: (1) analyze historical epidemiological information from the 2012 YFV outbreak to identify risk factors; (2) understand contemporary mosquito habitats and species distribution, and to perform molecular screening for circulating arboviruses; and (3) determine current knowledge and attitudes toward YF within the affected communities and assess community-level practices for YF prevention.

## Materials and Methods

### Study location

**Figure 1:**
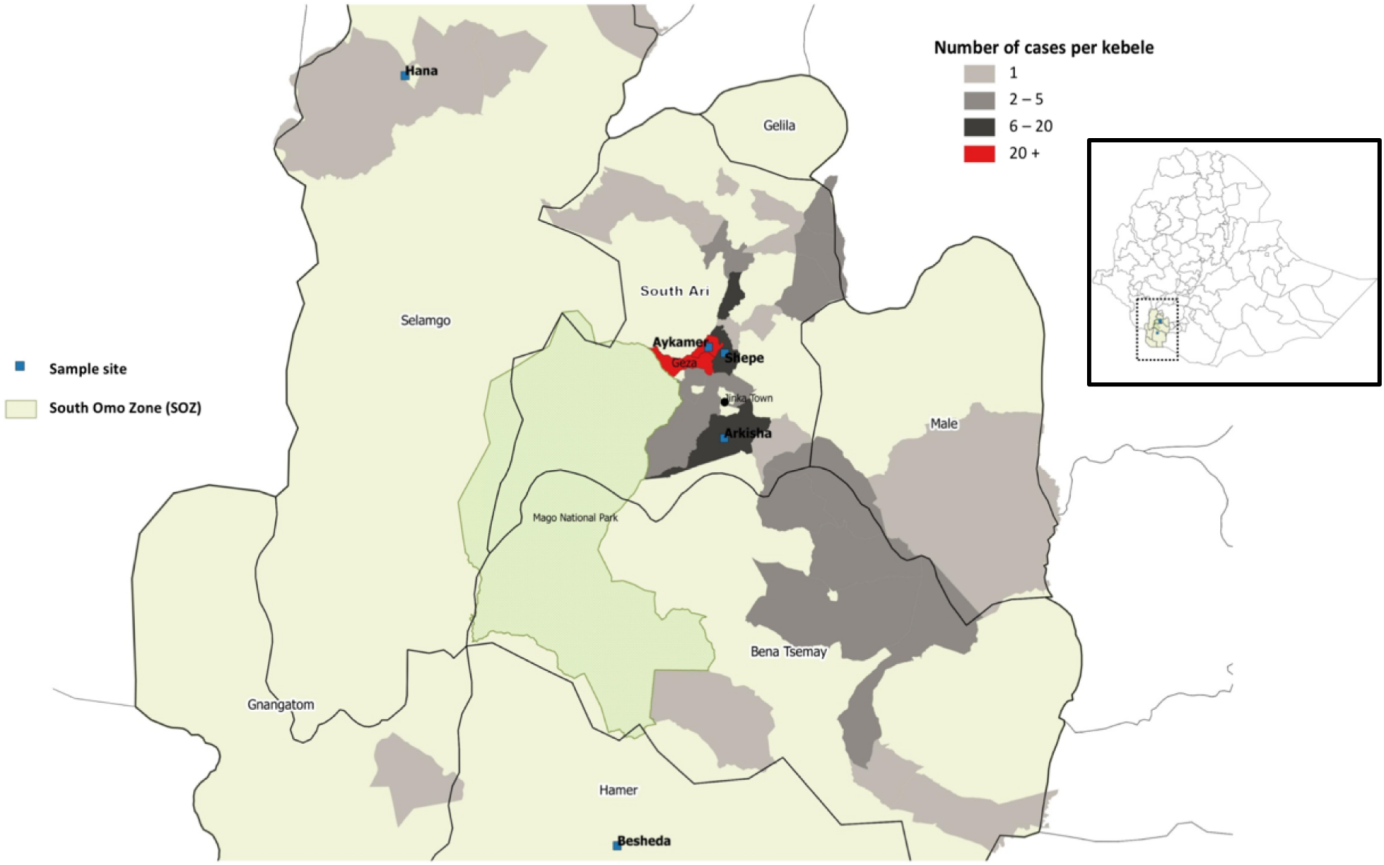
Map of study sites in South Omo Zone (SOZ), Southern Nations Nationalities and People’s Region (SNNPR), South-West Ethiopia, showing distribution and frequency of YFV cases (*n* = 165).

The study was conducted in South Omo Zone (SOZ), Ethiopia, which is located in South-West Ethiopia (Southern Nations Nationalities and People’s Region—SNNPR) (Fig. 1) and consists of 16 different tribes, including nomadic groups. Average temperature of the zone ranges from 10.1 °C to 27.5 °C, with a mean annual rainfall ranging from 400 to 1,600 mm; the main rainy season occurs from March to September. There are two national parks situated inside SOZ—Omo National Park and Mago National Park—in addition to a number of smaller forest areas. The total population of SOZ in 2016 was 731,805. South Ari is the most populated woreda (region) with a total population of 237,988 in 50 kebeles (47 rural and three urban villages). Five kebeles were selected for the study—Aykamer, Shepe, Arkisha (South Ari woreda), Hana (Salamago woreda), and Besheda (Hammer woreda), which reported varying numbers of cases during the 2012–2014 outbreak and were all targeted for vaccination during the emergency reactive campaign in 2013.

### Historical clinical data collection

In November 2012, cases of an unknown febrile illness were reported to the Public Health Emergency Management (PHEM) through the mandatory weekly reporting format for Health Extension Workers (HEWs) on all immediately reportable diseases. Symptoms reported by cases were a combination of fever, headache, nausea, bloody vomiting, abdominal pain, joint pain, and jaundice. A team from the SOZ Health Department, World Health Organization (WHO) Ethiopia Country Office and Ethiopian Public Health Institute (EPHI) was deployed to the field for a rapid risk assessment. The causative agent was confirmed as YFV, following laboratory testing for IgM ELISA (and PRNT for differential diagnosis) in the regional reference laboratory for YFV (Institut Pasteur, Dakar, Senegal). A WHO standard case definition was used during the field investigation (World Health Organization (WHO), 2003) to define suspected cases; in addition epi link was used by HEWs. The line list of all suspected and confirmed cases during the outbreak was retrieved from the SOZ Health Department in June 2017, to conduct a full historical descriptive epidemiological analysis; an interim report detailing suspected and confirmed cases only up to October 2013 was reported by Lilay et al. (2017).

### Entomological investigation and sampling

At each household premise selected to participate in the Knowledge, Attitudes, and Practices (KAP) survey (described below), mosquito larvae and pupae were sampled from all natural and artificial containers found around the home using a dipper. Only containers holding water were included. An entomological survey was completed to define the type of container, its location, its usage and larval and pupal densities to assess the most productive breeding sites. Any container that was found harboring at least one larva or pupa of *Aedes* spp. was considered positive, and a sample of all immature stages collected were reared to adulthood for subsequent morphological identification (Hopkins, 1952; Ward, 1981). The following density indices were calculated:

House index (HI)—the percentage of houses found positive for mosquito larvae or pupae.
Container index (CI)—the percentage of containers found positive for mosquito larvae or pupae.
Breteau index (BI)—the number of containers found positive for mosquito larvae and pupae per 100 houses surveyed.
Pupal demographic index (PDI)—number of pupae found per number of residents in the houses inspected.

Entomological indices were interpreted according to the WHO guidelines; a high risk of YFV transmission is HI > 35%, BI > 50, or CI > 20% and low risk is HI < 4%, BI < 5, or CI < 3% (World Health Organization (WHO), 1971). The most productive containers were considered the types of water-holding containers where >70% of all pupae were found (Focks, 2003).

A Prokopack aspirator (Vazquez-Prokopec et al., 2010) was used to collect adult mosquitoes at each household, both inside and outside of the premises. Each location was sampled for a total of 15 min (50% inside, 50% outside) to ensure systematic sampling. Specimens were labelled with date, time and location of their collection. Captured adult mosquitoes were identified in the field as *Aedes* spp. or non-*Aedes* spp. by their taxonomic features (Hopkins, 1952). A total of 90% of the adult female *Aedes* mosquitoes were stored in RNAlater^®^ (AM7020; Sigma, Gillingham, UK) at 4 °C or lower to prevent viral RNA degradation, while the remaining 10% of *Aedes* samples were stored dry for molecular species identification.

### Molecular identification of mosquito species

RNA was extracted from individual whole mosquitoes using QIAGEN RNeasy 96 kits (74182) according to manufacturer’s instructions. RNA was eluted in 40 μL of RNase-free water and stored at −80 °C. A QIAGEN QuantiTect Reverse Transcription kit (205313) was used to reverse transcribe RNA to generate cDNA from all RNA extracts according to the manufacturer’s instructions. To determine the species of adult female *Aedes* collected in Shepe, Aykamer, and Arkisha, a fragment of the ITS2 gene was sequenced (Beebe & Saul, 1995). PCR products were separated and visualized using 2% E-Gel EX agarose gels (G402002; Invitrogen, Carlsbad, CA, USA) with SYBR safe and an Invitrogen E-Gel iBase Real-Time Transilluminator. PCR products were submitted to Source BioScience (Source BioScience Plc, Nottingham, UK) for PCR reaction clean-up, followed by Sanger sequencing to generate both forward and reverse reads. Sequencing analysis was carried out in MEGA7 (Kumar, Stecher & Tamura, 1874). Both chromatograms (forward and reverse traces) from each sample were manually checked, analyzed, and edited as required, followed by alignment using ClustalW and checking to produce consensus sequences. Consensus sequences were used to perform nucleotide BLAST (NCBI) database queries and an alignment constructed to include all field sample consensus sequences; two consensus sequences from *A. bromeliae* specimens, generated by following the same procedure as the field samples, plus relevant sequences covering the region sequenced, were obtained from GenBank. This alignment by ClustalW was used to produce a Maximum likelihood phylogenetic tree based on the Tamura–Nei method (Tamura & Nei, 1993).

### Arbovirus screening

Screening for YFV and other major arboviruses of public health suspected or having the potential of being transmitted in the region (Dengue virus (DENV), Zika virus (ZIKV), Chikungunya virus, West Nile virus (WNV), and Rift Valley Fever virus) was undertaken with collected adult female *Aedes* spp. mosquitoes (samples stored in RNAlater^®^ as described above) using published real time PCR assays (File S1). No immature species were screened. PCR reactions for all assays except ZIKV and WNV were prepared using five μL of QIAGEN QuantiNova SYBR Green Master mix (208052), a final concentration of one μM of each primer, one μL of PCR grade water and two μL template cDNA, to a final reaction volume of 10 μL. Prepared reactions were run on a Roche LightCycler^®^ 96 System and PCR cycling conditions are described in File S1. Amplification was followed by a dissociation curve (95 °C for 10 s, 65 °C for 60 s and 97 °C for 1 s) to ensure the correct target sequence was being amplified. ZIKV and WNV screening was undertaken using a Taqman probe-based assay using five μL of QIAGEN QuantiTect probe master mix (204345), a final concentration of one μM of each primer, one μL of PCR grade water and two μL template cDNA, for a final reaction volume of 10 μL. PCR results were analyzed using the LightCycler^®^ 96 software (Roche Diagnostics, Risch-Rotkreuz, Switzerland). Synthetic long oligonucleotide standards (Integrated DNA technologies, Coralville, IA, USA) of the PCR product template sequence were generated in the absence of biological virus cDNA positive controls and each assay included negative (no template) controls.

### Knowledge, attitudes, and practices study design and sample population

A total of 180 households participated in the KAP survey across the five sites during the study period. A full household list was gathered from each village health post and random sampling used to select households, using a random number generator. Study inclusion criteria included the ability of the respondent to give informed consent and residency in the area of the 2012–2014 outbreak at that time. Sample size calculations were conducted in STATA/IC 14.2 using the *svysampsi* command for surveys with a dichotomous outcome variable, with a proportion of 0.9 assumed to have the expected outcome (knowledge of YF), an error rate of 5%, a response rate of 90%, and a 95% confidence interval.

The KAP survey was developed through adaptation of previous KAP surveys used for other arboviruses (DENV and ZIKV), both in-country and from other endemic regions, as well as compilation of context-specific questions following informal discussions with stakeholders in Ethiopia (Shuaib et al., 2010; Dhimal et al., 2014; World Health Organization (WHO), 2016b). The survey was semi-structured, including both open and closed-ended questions, and captured details on household characteristics (socio-economic status/education), past YFV infection, self-reported vaccination status and general *Aedes* spp. control practices (File S2). The survey was structured into eight main sections: (1) socio-demographic characteristics of study participants; (2) knowledge of YF symptoms, signs and transmission modes; (3) attitudes toward YF; (4) preventative practices against YF; (5) sources of information regarding YF; (6) YF case finding; (7) YF self-reported vaccination coverage estimation; and (8) other epidemiological risk factor information. The full questionnaire was first developed in English, translated into Amharic and re-translated back into English for analysis. Before its use in the study, the questionnaire was pilot tested among community members in Arkisha, who were not included in the final analysis.

### KAP data collection and analysis

At each study site, the questionnaire was conducted in Amharic (or local dialect if preferred by respondent) by a HEW, who was trained by an experienced interviewer. The head of household was the main respondent, however when this was not possible, another member of the family was interviewed *in lieu*. All completed household surveys were double-checked and verified on the same day for completeness and consistency. Data were interpreted taking into account informal conversations and observations in the community and hospital.

The KAP assessment was conducted using a scoring system. A participant’s KAP score was calculated as the sum of their correct answers, where a correct answer was given a value of 1 and an incorrect answer (including any answers “do not know” or “no answer”) the value of 0 (Dhimal et al., 2014). The total possible score was 15 for Knowledge, five for Attitude and eight for Practices. Respondents’ levels were defined as “good” or “poor” based on a 75% cut-off threshold. Logistic regression was conducted in STATA/IC 14.2 to identify determinants for KAP levels. Independent variables included in the model were household location, YF vaccination status, education level and the age and sex of respondent.

### Ethical approval

Ethical approval for the study was obtained from the London School of Hygiene and Tropical Medicine (LSHTM; ref #12291) and Arba Minch University (AMU; ref #6008/111) and all study procedures were performed in accordance with relevant guidelines and regulations. An invitation letter from AMU was taken to SOZ Health Department, who then provided an invitation letter, which was delivered to the village health posts to gain permission to sample. All participants were adults and gave full written informed consent. Confidentiality of all respondents was assured by using unique study identifiers. Working closely with HEWs ensured community acceptance and cooperation.

## Results

### Epidemiological investigation

**Figure 2:**
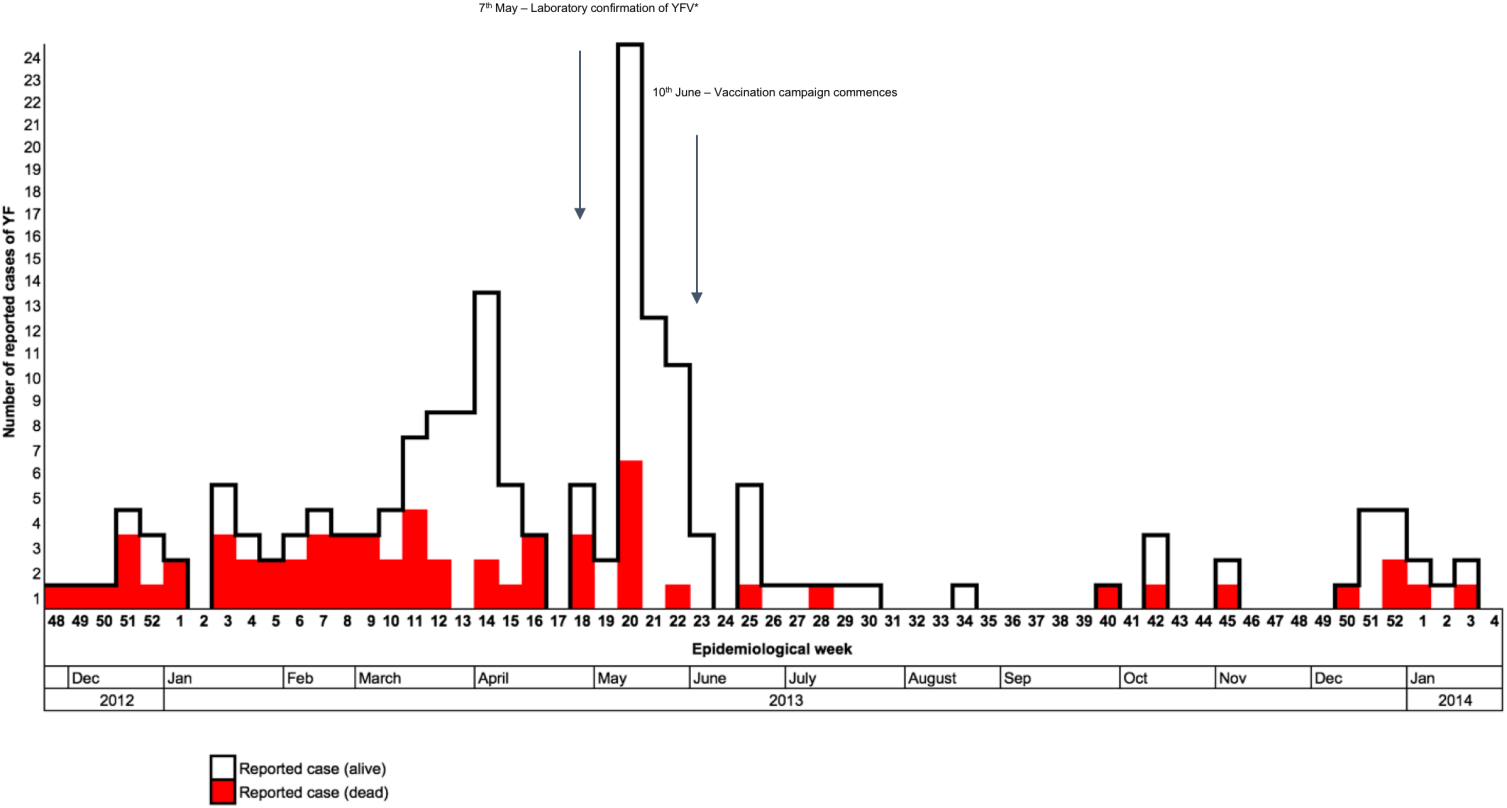
Distribution of reported yellow fever cases by their date of onset, from November 2012 to January 2014 (*n* = 165) in Southern Nations, Nationalities, and People’s Region (SNNPR), Ethiopia. *YFV, Yellow fever virus.

Between November 2012 and March 2014, a total of 165 suspected cases of YFV were reported to the PHEM, including 62 deaths. Of these cases, six had IgM laboratory confirmation from the regional reference laboratory (Institut Pasteur, Dakar, Senegal), with the remaining 159 cases defined through epi-link. The index case was reported from Geza, with onset of symptoms between November 12 and 23, 2012. The index case was laboratory confirmed on May 7, 2013. The main peak of the outbreak occurred from March to May 2013, with cases appearing to decline following the emergency vaccination campaign which commenced on June 10, 2013 (Fig. 2).

The majority of cases were 15–44 years old (75.8%; AR: 5.3 per 10,000), with males slightly more affected than females, with a male to female case ratio of 1.4:1 (Tables 1 and 2). Most case deaths were also in males, which resulted in a case fatality rate (CFR) of 48.5% in males compared to 22.1% in females (Table 3). The overall CFR was 37.6%, with the majority of deaths occurring at the start of the outbreak. A total of 69 percent of reported deaths occurred in health facilities; the remaining 31.0% were community deaths found through active case detection.

**Table 1:** Age, sex, and residence of reported YFV cases during the outbreak in Southern Nations, Nationalities, and People’s Region (SNNPR), Ethiopia (*n* = 165).

	Variable	Number of suspected cases	Percentage (%)
Age	0–4	5	3.0
	5–14	16	9.7
	15–44	125	75.8
	45+	19	11.5
Sex	Male	97	58.8
	Female	68	41.2
Residence	South (Debub) Ari*	123	74.6
	BenaTsemay	19	11.5
	Jinka town	11	6.7
	Salamago*	3	1.8
South Omo Zone (SOZ)	Malle	3	1.8
	Gnangatom	1	0.6
	Dasenech	1	0.6
	North (Semen) Ari	0	0
	Hammer*	0	0
	Total	161	97.6
	Konso	2	1.2
Gamo Gofa Zone	Geze Gofa	1	0.6
	Mirab Abaya	1	0.6
	Total	4	2.4

**Note:**

* Contains one or more sampled kebele from this study.

**Table 2:** Age-specific YFV attack rates (ARs) as of March 2014 in Southern Nations, Nationalities, and People’s Region (SNNPR), Ethiopia (*n* = 165).

Age	No. cases	Population (2007)	AR (per 10,000)
0–4	5	100,503	0.5
5–14	16	183,816	0.9
15–44	125	238,221	5.3
45+	19	50,895	3.7

**Table 3:** Status of YFV patients as of March 2014 by sex, outcome status, and respective case fatality rate (CFR) in Southern Nations, Nationalities, and People’s Region (SNNPR), Ethiopia (*n* = 165).

	No. alive (%)	No. dead (%)	CFR (%)
Female	53 (51.5)	15 (24.2)	22.1
Male	50 (48.5)	47 (75.8)	48.5
Total	103	62	37.6

A total of 75.0% of all cases were reported from rural areas in South Ari. Aykamer, Geza, and Shepe kebeles contributed 49.0% of all reported cases and 62.0% of reported cases within South Ari woreda (Table 1; Fig. 2). Aykamer and Geza had the highest attack rates (AR) at 103.2 and 104.7 per 10,000 population, respectively. On June 10, 2013 the SOZ Health Department began an emergency vaccination campaign which targeted 607,462 people (World Health Organization (WHO), 2016a). HEWs and allied health professionals were able to vaccinate 543,558 people and an overall coverage estimate of 89.0% was reported by the SOZ Health Department in March 2014.

### Entomological investigation

A total of 688 containers containing water were inspected among 180 households between June and August 2017 (rainy season). Overall, 240 (34.9%) of the containers were classified as positive, that is, contained at least one mosquito larva or pupa, across a total of 105 positive households (59.3%). The majority of water-filled containers (including artificial containers) were found outdoors and filled with rainwater. The locations and types of containers across the kebeles varied, reflecting the differences in larval indices among study sites. No larvae or pupae were found in any water-holding containers in Besheda. A sample of all immature stages were reared to adulthood (Table 4). From Shepe, Arkisha, and Aykamer, the main species collected was *Aedes* spp., however from Hana a large proportion was *Culex* spp. A small number of *Toxorhynchites* spp. larvae and pupae were identified in Shepe and Aykamer.

**Table 4:** Species of mosquitoes from immature stages reared to adults across sample sites.

	Shepe	Arkisha	Aykamer	Hana	Besheda
*Aedes* spp.	44 (76%)	35 (90%)	84 (76%)	5 (19%)	0 (0%)
*Culex* spp.	12 (21%)	4 (10%)	23 (21%)	22 (81%)	0 (0%)
*Toxorhynchites* spp.	2 (3%)	0 (0%)	3 (3%)	0 (0%)	0 (0%)
Total	49	37	110	27	0

**Figure 3:**
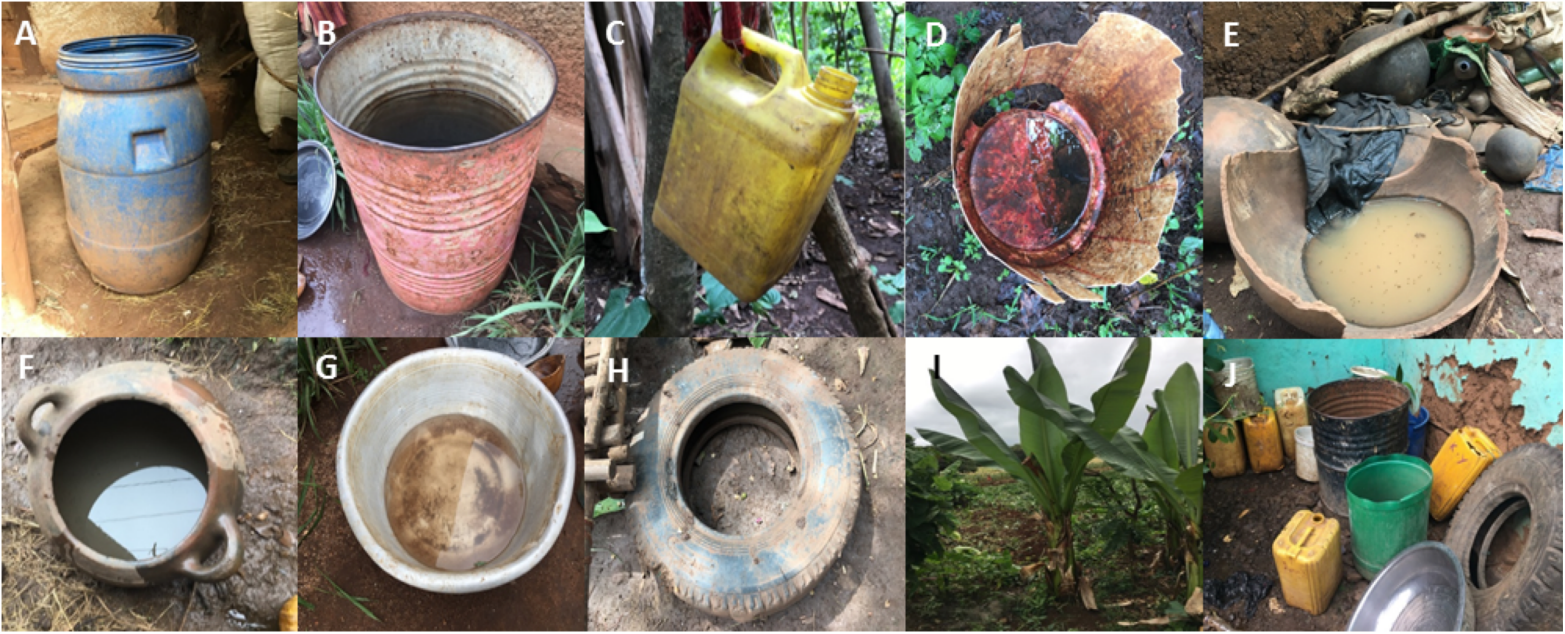
Typical mosquito breeding sites identified in South Omo Zone, Ethiopia, 2017: (A) Plastic drum (B) Metal drum (C) Plastic jug (D) Discarded plastic (E) Discarded clay pot (F) Clay pot (G) Metal bowl (H) Discarded Tire (I) False banana plant (J) Other. Image credit: Ranya Mulchandani.

In the highland area of South Ari, the false banana plant (*Ensete ventricosum*) is ubiquitously found in close proximity to the home (<10 m) and was the major site for immature *Aedes* spp. stages in Shepe (75% of plants inspected were positive) and Aykamer (64% positive). The second most important breeding sites in these two kebeles were discarded plastic and clay, which were commonly observed outside the home (Fig. 3). There was also a high coverage of mixed vegetation close to the home, including sweet potato, maize, and false banana, which were often the locations of adult mosquito collections. By comparison, the homes in Besheda were frequently kept completely clear of any containers, clutter or vegetation. It was very unusual to see any containers outside of the home. The types of infested water-holding containers varied between the sample sites depending on local usage and traditional practices (Table 5).

**Table 5:** Different types of containers inspected, including proportion found positive with either mosquito larvae or pupae, and relative pupal contribution, across sample kebeles in South Omo Zone, Ethiopia, 2017.

	Type of container	No. of containers inspected	No. of positive containers* (%)	No. of pupae (% contribution)
Natural breeding sites	*Ensete ventricosum* (False banana)	220	141 (64.2)	179 (19.7)
	*Musa* spp. plant (Banana)	28	18 (64.3)	19 (2.1)
	Tree hole or tree trunk	6	3 (50.0)	27 (3.0)
	*Colocasia esculenta* (Taro)	1	0 (0.0)	0 (0.0)
Artificial breeding sites	Plastic jug	101	5 (4.9)	3 (0.3)
	Plastic drum/metal drum	110	8 (7.3)	51 (5.6)
	Discarded plastic	65	14 (21.5)	86 (9.5)
	Discarded clay pot	48	9 (18.8)	304 (33.5)
	Clay pot	45	10 (22.2)	225 (24.8)
	Plastic bowl	23	0 (0.0)	0 (0.0)
	Metal bowl	27	2 (7.4)	3 (0.3)
	Discarded tire	7	4 (57.1)	8 (0.9)
	Discarded plastic shoe	4	2 (50.0)	2 (0.2)
	Water pump	1	1 (100.0)	0 (0.0)
	Discarded metal	1	0 (0.0)	0 (0.0)
	Glass bottle	1	1 (100.0)	0 (0.0)
Total		688	34.9	907 (100)

**Note:**

* Presence of at least one larva and/or pupa.

Table 6 shows the BI, CI, PDI, and HI for each study site. The highest HIs were recorded in Shepe (79.0%) and Hana (57.1%); both villages were classified as high risk for YFV transmission (WHO threshold of >35%). The highest CIs were in Shepe (57.9%) and Aykamer (75.4%), both of which inferred a high risk (CI > 20%). The BIs were higher than the WHO threshold across all sample sites except Besheda (threshold of BI > 50); indicating there is no evidence for YFV transmission in Besheda as no immature stage was found and all indices were below the low risk thresholds of HI < 4%, CI < 3%, and BI < 5. The PDI was highest in Aykamer (2.24), which also had the highest AR (103.2 cases per 10,000 people) during the 2012–2014 outbreak (World Health Organization (WHO), 1971). There was no statistically significant correlation between traditional entomological indices and AR. However, there was strong positive correlation (*r* = 0.9545) between AR and PDI (*p* = 0.0455).

**Table 6:** Distribution of entomological indices and YFV attack rates recorded across sample kebeles, South Omo Zone, Ethiopia, 2017.

Woreda	Kebele	Total houses inspected	Household index (%)	Container index (%)	Pupal demographic index*	Breteau index	YFV attack rate^
South Ari	Shepe	43	79.0	57.9	0.48	237.2	35.5
	Arkisha	26	38.5	18.5	0.28	76.9	9.8
	Aykamer	65	33.8	75.4	2.24	144.6	103.2
Salamago	Hana	21	57.1	24.0	0.50	114.3	3.2
Hammer	Besheda	25	–	–	–	–	–
	Total	180	41.7	35.2	0.70	114.6	30.3

**Notes:**

* Number of pupae per person.

^ Number of cases per 10,000 people.

**Figure 4:**
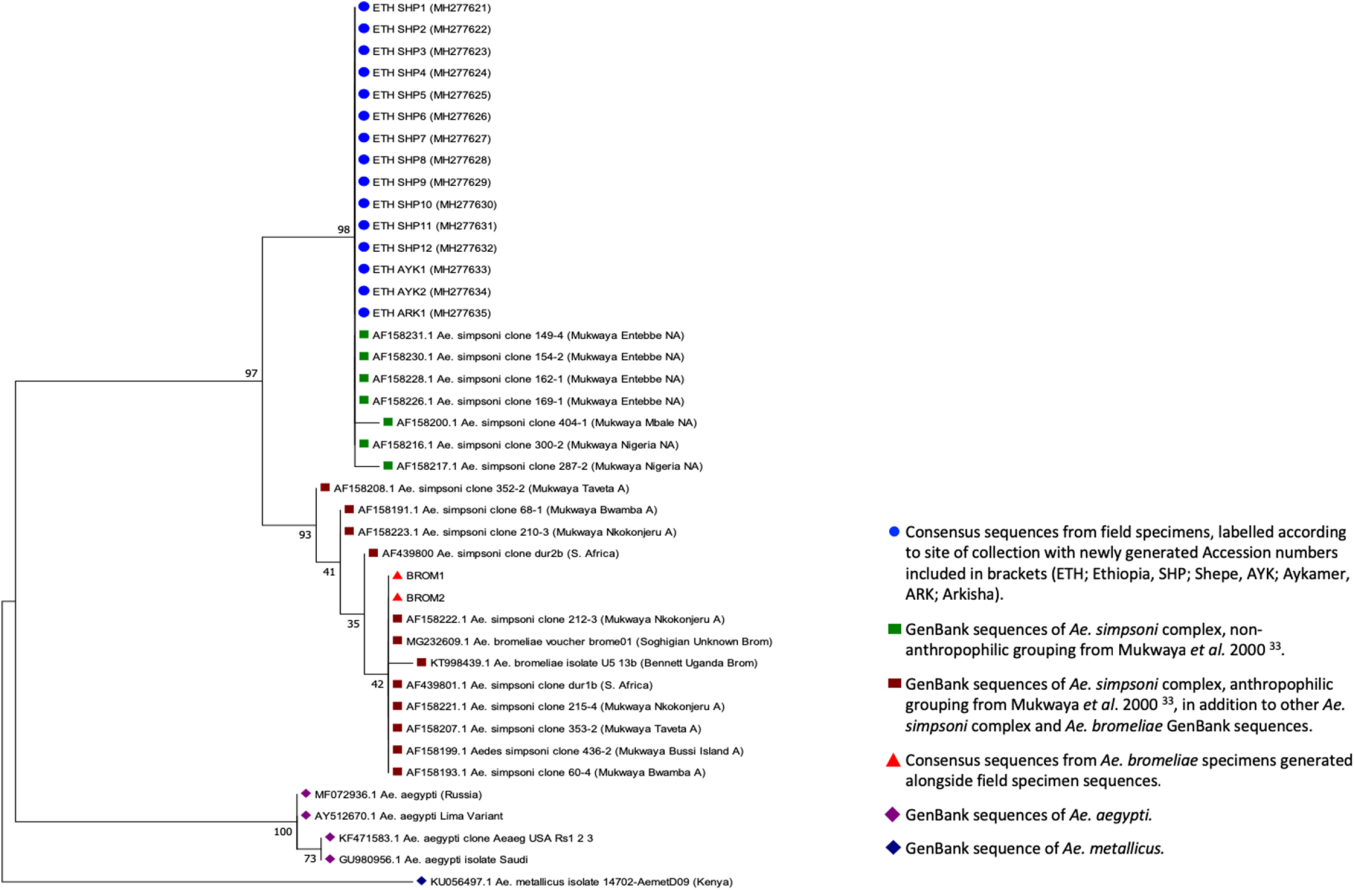
ITS2 phylogenetic analysis by Maximum Likelihood method. The evolutionary history was inferred using the Maximum Likelihood method based on the Tamura–Nei model. The tree with the highest log likelihood (−587.45) is shown. The percentage of trees in which the associated taxa clustered together is shown next to the branches. Initial tree(s) for the heuristic search were obtained automatically by applying Neighbor-Joining and BioNJ algorithms to a matrix of pairwise distances estimated using the Maximum Composite Likelihood (MCL) approach, and then selecting the topology with superior log likelihood value. The tree is drawn to scale, with branch lengths representing the number of substitutions per site. The analysis involved 41 nucleotide sequences. Codon positions included were 1st+2nd+3rd+Noncoding. All positions containing gaps and missing data were eliminated. There was a total of 230 positions in the final dataset. Evolutionary analyses were conducted in MEGA7.

Screening individual adult female *Aedes* mosquitoes collected using aspiration (*n* = 120 from Shepe, *n* = 12 from Aykamer, *n* = 1 from Arkisha) for YFV and other major arboviruses revealed no evidence of medically-important arbovirus infection. Molecular species identification was undertaken by sequencing a fragment of the ITS2 gene for a sub-sample of specimens (approximately 10% of the total number from each location) which included 12 individuals from Shepe, where the majority of adult females were collected, two from Aykamer and one from Arkisha, in addition to two *A. bromeliae* specimens from Tanzania for comparison. Figure 4 shows that all specimens collected in our study were phylogenetically similar to GenBank sequences from the *A. simpsoni* complex, non-anthropophilic grouping from Mukwaya et al. (2000), indicating these specimens are part of the *A. simpsoni* complex and group with *A. lilii.* Consensus sequences from this study have been submitted to GenBank (accession numbers MH277621—MH277635).

### Household and individual characteristics

The KAP respondents comprised 101 (56.1%) females and 79 (43.9%) males, with 111 (62.3%) between 20–34 years of age (Table 7), sampled from the same households which participated in the entomological survey. The majority either had no education or primary school level (86.6%), while only 5.6% had achieved a higher education level. Among all respondents, 142 (78.9%) were farmers, while the remaining 38 (21.1%) were a combination of other professions.

**Table 7:** Demographic characteristics of study respondents to KAP household questionnaire in South Omo Zone, Ethiopia (*n* = 180).

	Variable	Frequency	Percentage %
Sex	Male	79	43.9
	Female	101	56.1
Age (years)	<20	5	2.8
	20–24	23	12.8
	25–29	39	21.7
	30–34	50	27.8
	35–39	33	18.2
	>40	30	16.7
Education	None	96	53.3
	Primary school	60	33.3
	Secondary school	14	7.8
	Higher education	10	5.6
Marital status	Married	152	84.4
	Single	12	6.7
	Divorced	2	1.1
	Widowed	14	7.8
Occupation	Unemployed	14	7.8
	Farmer	142	78.9
	Shopkeeper	1	0.6
	Forest-worker	1	0.6
	Trader	4	2.2
	Teacher	3	1.6
	Student	6	3.3
	Other	9	5.0
No. of people in the household	<4	26	14.4
	4	43	23.9
	5	29	16.1
	6	40	22.2
	7	19	10.6
	>7	23	12.8

### Knowledge of YF

A total of 158 (87.8%) of the respondents had previously heard of YF, with the majority able to identify the general symptoms of YF, including fever (82.2%) and headache (82.2%) (File S3). Fewer participants were able to identify more specific YF symptoms, including jaundice (61.7%), muscle pain (74.5%), and bloody vomiting (56.1%). Most participants stated that mosquitoes transmitted YFV (82.8%) (File S3). Many respondents (57.2%) believed that YFV could be transmitted through ordinary person to person contact, although knowledge was higher that it is not possible to transmit YFV via food or water (62.8%). Many thought YFV mosquitoes were most likely to bite at night time (58.9%). The majority of respondents knew that mosquitoes could breed in standing water (83.9%) but much fewer were aware that this was possible inside the home (46.7%). A total of 138 (76.7%) householders agreed that removing or covering standing water helps to prevent mosquito breeding and 145 (80.6%) agreed that pouring chemical into standing water was effective at killing mosquito larvae.

### Attitudes toward prevention and control of YF

A total of 147 (81.7%) of study respondents stated that YF is a serious illness, citing such reasons as bloody vomiting, high CFR, and becoming more dangerous without early treatment (File S4). Respondents agreed that both controlling breeding sites of mosquitoes (87.2%) and vaccination (75.6%) were good strategies to prevent YF, and that communities have a role to play in controlling these mosquitoes (86.7%). Fewer participants (56.1%) thought that it was the health post’s responsibility to prevent YF.

### Practices regarding YF prevention

The majority of respondents (92%) self-reported actively reducing mosquitoes near their homes, through preventing standing water (89.4%), using insecticide-treated nets in the home (85.6%), and using smoke to drive mosquitoes away (86.7%); in addition, the majority reported wearing clothes to prevent mosquito bites (92.8%) (File S5). A total of 155 (81.0%) respondents reported that the government had come to their houses to spray insecticides on their walls in the past. Most individuals reported covering water containers in the home (90.0%), with many claiming to clean their water-filled containers every day (37.3%) or at least once a week (51.7%) and then turning their containers upside down to avoid water collection (84.4%).

### Sources of information about YF

The majority of respondents (76.8%) reported receiving information solely from their HEWs, particularly as most lived in rural kebeles without access to radio or television. A large proportion of respondents knew of neighbors or family members who had YF in the past, with many describing cases that occurred 5–6 years ago, that is, during the 2012–2014 outbreak. However, individuals often cited cases occurring after the outbreak (which was considered over in late 2014), particularly in Arkisha where 64.0% of respondents reported knowing someone who had YF in 2016 or 2017. A total of 60% of respondents from Arkisha self-reported having suffered from YF themselves, while no one in Besheda reported any cases of YFV for either themselves or anyone else they knew.

Self-reported vaccination history, collected alongside household KAP survey data, reported only 62.0% of respondents having been vaccinated in the YFV campaign in 2013, compared to the 89.0% coverage estimated by SOZ Health Department by March 2014. All of those who reported being vaccinated, had done so at their kebele health post in 2013.

A total of 21 (11.7%) study respondents reported working in Mago National Park or other forest areas, particularly from Arkisha which borders the park. A total of 45 (25.0%) people reported having contact with monkeys near their homes. In Hana, almost every respondent (90.5%) had recently moved to the region for work (within the last 5–10 years), in particular to work at the Omo Kuraz Sugar Factory project, or to serve this growing urban community.

### Predictors for knowledge, attitudes, and practices

Regarding KAP scores, 53.3% of participants achieved a good (defined as at least 75% of questions answered correctly) knowledge score, 78.9% achieved a good attitude score and 70.6% a good practices score. In the univariate analysis of the association between KAP score and a number of independent variables (including socio-economic variables, past YFV infection and YF vaccination status), there was increased odds of having good knowledge if the respondent lived in Arkisha (OR:13.88; 95% CI [4.18–46.02]), Aykamer (OR: 18.89; 95% CI [6.74–52.94]), Hana (OR: 7.40; 95% CI [2.22–24.65]), and Besheda (OR:5.22; 95% CI [1.61–16.95]), compared to a respondent living in Shepe (Table 8). This analysis also predicted increased odds of having a good attitude score if the respondent was female (OR: 2.71; 95% CI [1.29–5.67]), and a decreased odds of good attitude by lack of YF vaccination (OR: 0.40, 95% CI [0.19–0.83]) (Table 8). A good practice level score was also found to be predicted by YF vaccination status (OR: 0.37, 95% CI [0.19–0.71]). Age was not found to be a predictor for knowledge, attitudes, or practices. After adjusting for potential confounders in the multivariate analysis, sex was no longer found to be a significant predictor of attitude levels; or vaccination status on knowledge level (Table 8).

**Table 8:** Logistic regression analyses showing predictors of knowledge, attitude, and practices levels (good vs. bad).

Dependent variable	Independent variable	Categories	OR^+^ (95% CI)	aOR* (95% CI)	*p*-Value^
Knowledge level	Vaccination status	Yes	1	1	
		No	1.17 [0.64–2.16]	0.60 [0.26–1.39]	0.231
	Household location	Shepe	1	1	
		Arkisha	13.88 [4.18–46.02]	48.97 [9.40–255.12]	<0.001
		Aykamer	18.89 [6.74–52.94]	31.97 [8.15–120.62]	<0.001
		Hana	7.40 [2.22–24.65]	25.35 [4.75–135.46]	<0.001
		Besheda	5.22 [1.61–16.95]	9.71 [2.07–45.53]	0.004
Attitude level	Sex	Male	1	1	
		Female	2.71 [1.29–5.67]	2.33 [0.79–6.84]	0.124
	Vaccination status	Yes	1	1	
		No	0.40 [0.19–0.83]	0.21 [0.06–0.68]	0.009
	Household location	Shepe	1	1	
		Arkisha	3.33 [1.05–10.46]	14.03 [2.52–78.25]	0.003
		Aykamer	9.50 [3.18–28.34]	27.85 [5.17–150.03]	<0.001
		Hana	1.14 [0.40–3.24]	2.19 [0.46–10.34]	0.324
		Besheda	–	–	–
Practice level	Vaccination status	Yes	1	1	
		No	0.37 [0.19–0.71]	0.34 [0.15–0.80]	0.014
	Household location	Shepe	1	1	
		Arkisha	0.44 [0.14–1.40]	0.95 [0.23–3.84]	0.941
		Aykamer	0.26 [0.10–0.66]	0.26 [0.08–0.80]	0.019
		Hana	0.42 [0.12–1.40]	0.98 [0.20–4.80]	0.979
		Besheda	1.36 [0.32–5.84]	2.07 [0.41–10.54]	0.382

**Notes:**

OR, odds ratio.

aOR, adjusted odds ratio.

CI, confidence intervals.

+ Univariate analysis.

* Multivariate analysis adjusted for age, sex, marital status, education level, vaccination status, and household location.

^ *p*-Value corresponds to multivariate analysis.

The correlation of KAP scores revealed a slight positive correlation between knowledge and attitude scores (*r* = 0.41, *p* < 0.001); while no significant correlation was found between knowledge and practices (*p* = 0.373), or attitude and practices (*p* = 0.471) (Table 9).

**Table 9:** Correlation between knowledge, attitude, and practices scores.

Variables	Correlation co-efficient (*r)*	*p*-value
Knowledge-attitudes	0.41 (0.24, 0.58)	<0.001
Knowledge-practices	−0.07 (−0.23, 0.09)	0.373
Attitudes-practices	0.05 (−0.08, 0.18)	0.471

## Discussion

To reduce the risk of future YFV and other arboviral disease outbreaks through appropriate interventions and policy recommendations, it is important to understand past outbreaks and community perceptions and preventative practices in at-risk areas. From 2012 to 2014, 165 YFV cases were reported in SOZ, including 62 fatalities. YF was unfamiliar to health professionals at that time as it was an unexpected outbreak of a disease that had not been seen locally since 1962 (Serie et al., 1964). With the majority of cases identified in rural kebeles, it is likely that the case numbers in the outbreak was underreported, while the severity might have been over-reported due to severe cases being more probable of being detected and reported. In addition, due to limited resources, the majority of cases were defined through epi-link (rather than laboratory confirmation) and there is a possibility that some of these cases may have been due to other circulating pathogens.

The epidemiological data indicate that the case number was higher in males, which is in line with previous YFV outbreaks, such as the 1960 outbreak in Ethiopia where the male:female case ratio was 1.6:1 (Serie et al., 1968) as well as in Uganda (2011) where the sex-specific AR was 16.5 (male) vs. 9.6 (female) per 100,000 (Wamala et al., 2012). The overall male-to-female sex ratio may suggest the outbreak was a result of sylvatic transmission (due to males working on the forest periphery and being bitten during the day compared to females who are less likely to be exposed to the mosquito’s bite), in line with modelling by Faria et al. (2018), however further studies would be required to confirm this.

The majority of cases were seen in 15–44 year-old individuals (75.8% of cases) who may have been infected while working outdoors during the day and dusk, the peak mosquito biting times; a risk factor also described in Kenya (1992–1993) where 81% of cases were <40 years old (Reiter et al., 1998). The overall CFR in SOZ was 37.6%, which is higher than in the Darfur epidemic of 2012 (20.3%) and in Uganda in 2011 (24.9%) (Alhakimi et al., 2015). Only four cases were reported from outside of SOZ (Gamo Gofa, Konso, and Mirab Abaya), which may be from individuals visiting the region, for example, becoming infected at the weekly Saturday market in Jinka Town; rather than autochthonous YFV transmission. However, with reports of *Aedes* spp. in Gamo Gofa (Ardoin, Rodhain & Hannoun, 1976), the potential for local transmission is possible (as seen in 1966, a region previously unaffected during the 1960–1962 outbreak). Areas outside of SOZ were not included in the mass vaccination campaign of 2013, leaving this population immunologically-vulnerable to subsequent outbreaks such as that seen in 2018.

Following the mass emergency vaccination campaign of 2013, the number of cases declined and a high administrative vaccination coverage was reported (however there was a discrepancy observed between the administrative coverage and self-reported status which could be due to a multitude of reasons). The onset of the vaccination campaign coincided with the peak of the outbreak and therefore it is possible that increasing vaccination coverage, alongside other contributing factors, such as increasing immunity in the population through natural infection were responsible for curbing the outbreak. Cases were still appearing in early 2014: for example, three cases in Malle woreda (previously unaffected during the peak period of the outbreak) were informally reported in individuals who had not been vaccinated (Zemelak, 2014). The recent reports from South Omo of YF cases in 2018 highlight the urgent need for a follow-up vaccination campaign and strengthened surveillance and case investigation.

In early 2014, the EPHI conducted a training for health managers at regional and district levels on YF. However, by this time, the peak of the outbreak was already over. In addition, surveillance was slow, taking almost 8 months for laboratory confirmation of the index case and therefore to confirm the outbreak. These delays were due to a combination of a lack of in-country laboratory diagnostics (time taken for samples to be sent to the Regional Reference Laboratory at Institut Pasteur, Dakar for analysis), non-specificity of the case definition, and potential misdiagnosis or omission by clinicians (Lilay et al., 2017; Gardner & Ryman, 2015). Through informal discussions, it was noted that most health professionals (particularly doctors) working in Jinka Hospital rotate to new hospitals after 2–3 years. Many of the doctors currently at the hospital did not know the signs, symptoms or treatment for YF. Therefore, knowledge levels of healthcare professionals can be assumed to be low in the case of future outbreaks.

During the entomological investigations, a total of 688 artificial and natural containers were inspected from across 180 households. Most of the containers were found outside of the home, and due to the ongoing rains in South Ari, filled with rainwater. None of the kebeles sampled had piped water, so buckets and drums were often used for water storage. The urban area of Hana had a larger number of people per household, and therefore residents stored a larger quantity of water, increasing the number of potential sites for harboring immature stages. The main breeding site in Aykamer and Shepe was the false banana plant, a crop found ubiquitously in South Ari. Traditional practices ensure the plant is cultivated close to the home, particularly as it requires a larger amount of dung and nutrients than the regular banana plant. The plant has multiple purposes; its leaves are used to transport cabbage to market and its stem and roots are used as food (the “false banana” fruits are inedible). In addition, this plant grows for about 4 years, providing a longer life-span to act as a mosquito breeding site, in comparison to the banana plant which grows, fruits and dies within a few weeks. In the hotter and drier months, the water in the large plant stem has previously been reported to resist evaporation, making mosquito breeding possible all year round (Serie et al., 1964). A recent mini-drought in Arkisha resulted in the loss of all false banana plants, however, usually it is pervasive in this kebele. In addition, previous studies identified the taro plant as an important breeding site for *Aedes* spp. in East Africa, however, in this study it was often difficult to isolate water from the plant and only a small number were inspected, therefore its significance may be underestimated (Serie et al., 1964). Unfortunately, it is logistically unfeasible to target all natural breeding sites for vector control.

The greatest larval and pupal contribution across the study sites was from clay pots (both used and discarded) as well as the false banana plant. The clay pots were used for multiple purposes, but commonly found to store water/clay during house construction or alteration, or those that were broken and discarded containing rainwater; in the former, as the water was not being consumed, it was left uncovered. Drums were also often used for water storage; however, most were found negative. One study in Dire Dawa, Ethiopia, observed that artificial breeding sites (in particular tires and plastic drums) were the primary source of dengue vectors in an urban setting (Getachew et al., 2015; Woyessa et al., 2013). Various studies have found differing types of containers responsible for *Aedes* spp. breeding, such as discarded tires in Tanzania (Philbert & Ijumba, 2013), medium storage containers in Nigeria (Agwu, Igbinosa & Isaac, 2016) and natural sites in Kenya (Deming et al., 2016; Reiter et al., 1998). In our study, the majority of artificial sites were outside the home, filled with rainwater, in contrast with the findings of Lilay et al. (2017) who found most sites indoors. Therefore, it may be more feasible to design community awareness campaigns to reduce standing water in these artificial containers.

Larval entomological surveys were conducted to understand the larval density and mosquito abundance, to determine the future risk of YFV transmission. Both pupal demographic indices and standard larval indices were calculated, however their limitations of being only approximate measures of risk should be considered during their interpretation. In terms of entomological risk indices, all kebeles except Besheda were above the WHO high risk thresholds for one or more entomological index, indicating evidence for risk of local YFV transmission. As previous studies have reported a number of limitations associated with measuring larval indices, pupal numbers per person were also calculated (Focks & Chadee, 1997). The PDI was highest in Aykamer (2.24), and generally higher in rural areas, consistent with a previous study of *A. aegypti* breeding sites in Kenya (Midega et al., 2006). We observed a strong positive correlation between the AR and PDI, and therefore PDI should be considered a better predictor of YFV risk than standard larval indices.

The ITS2 sequencing data from adult *Aedes* captured in and around households in this study, in comparison with currently available sequences, indicated that they were most closely related to non-anthropophilic members of the *A. simpsoni* complex from Uganda and Nigeria (Mukwaya et al., 2000). This complex comprises three known species including *A. simpsoni* sensu stricto *A. lilii* and *A. bromeliae*, of which the latter is considered anthropophilic and has previously been incriminated as the principal vector of YFV epidemics in Ethiopia (Lilay et al., 2017; Rogers et al., 2011). Adult mosquitoes collected from SOZ were most likely *A. lilii* (*A. simpsoni* s.s. is confined to southern Africa) and therefore the absence of any medically important arboviruses which are transmitted through human blood-feeding would be expected in this species given no females have been recorded biting humans (Mukwaya et al., 2000). While *A. bromeliae* can breed across varied ecologies, *A. lilii* has been sampled from a more restricted range of plant axils (*Musa* spp. *Colocasia* spp. *Dracena* spp. and *Sansevieria* spp.) and not previously from inside the domestic environment (Huang, 1979, 1986). Additional sampling efforts are warranted to identify the vector species responsible for YFV transmission in South-West Ethiopia and its breeding patterns, to further define the local distribution and ecology of *A. lilii,* and in particular, the host feeding behavior of the species in this specific locality.

The KAP survey was completed by equal proportions of male and female respondents (43.9% and 56.1%, respectively) and the majority had very little education. Despite this, knowledge (53.3%), attitude (78.9%), and practice (70.6%) scores were surprisingly high across all respondents, which may also be explained by the confusion with, and fear of, malaria. The results from the study showed that 87.8% of respondents had heard of YF (Amharic translation: *bicha woba*). However, the translation of *bicha* = yellow and *woba* = malaria, meant the direct translation of *bicha woba* was “yellow malaria” which may have resulted in some confusion between YF and severe malaria. Knowledge levels of general symptoms were high, while the more specific symptoms were much lower, which is in line with the aforementioned confusion between diseases. Many individuals thought YFV could be transmitted from person to person, which could be an issue when people are seeking treatment and likewise when administering care to others. This finding is in agreement with a recent study by Legesse et al. (2018) that also reported that a fairly high percentage (55.9%) of respondents in SOZ believed YFV could be transmitted through breathing (Legesse et al., 2018). There were high knowledge levels that YFV was transmitted by a mosquito (82.8%), in contrast to Legesse et al. (2018), who found only 37.6% knew YFV was vector-borne; because awareness of malaria transmission by mosquitoes was high, the true knowledge level may have been overestimated in our study. Knowledge of malaria was not quantitatively assessed in this work, however, studies by Abate, Degarege & Erko (2013) in other parts of Ethiopia showed 85% of respondents to a malaria KAP survey stated its transmission to be by mosquitoes (Abate, Degarege & Erko, 2013). In our study, only 30.5% knew that the mosquito could bite during the daytime and therefore, many people may not be adequately protecting themselves from bites of *flavivirus* vectors. The majority of participants stated that YF was a serious illness, with reasons including bloody vomiting and problems with the brain, which is also in agreement with the potential confusion between YF and severe/cerebral malaria.

A number of respondents from Arkisha reported knowing someone who had suffered from YF in 2016 or 2017, or self-reporting having suffered from YF themselves. This is consistent with the reporting of 22 suspected cases in Arkisha in March 2016 by PHEM (Bekele, 2016). However, according to a Weekly Humanitarian Bulletin for Ethiopia in June 2016, these cases turned out to be negative following laboratory analysis (United Nations Office for the Coordination of Humanitarian Affairs (OCHA), 2016).

The majority of individuals received their information from HEWs, rather than television or radio. This is important knowledge to guide community awareness campaigns—for example, it may be preferable to educate HEWs with the appropriate information on YF to transfer this knowledge to their village, rather than through radio announcements. It is also important to consider methods for reaching those who are less likely to engage with their local health post in future YF educational campaigns. In addition, the finding of household location being predictive of the KAP scores was in agreement with how the respondents gathered their information on YF, with 76.8% reporting HEWs as their only source. This indicates that the odds of having good knowledge could partially be explained by how active and knowledgeable the HEWs were in the respective localities.

### Study limitations

There are several weaknesses in the reported study design, which need to be considered when interpreting the data. The line list of YF cases from the PHEM contained missing data on symptoms, occupation, travel history, or past vaccination history, and therefore, a clinical epidemiological analysis was not conducted and the scope for YF risk factor analysis was limited. As this analysis was retrospective and conducted after a recent vaccination campaign, it would not have been appropriate to perform a serological study to quantify asymptomatic infection rate, as it would not have been possible to differentiate between those who had received the vaccine from those with previous infection. However, future studies should consider incorporating serological surveillance to assess asymptomatic infection rate and more accurately measure CFR. Reports from other ecological regions in Ethiopia indicate low seroprevalence of actively circulating *flaviviruses* nationally, characterized by unpredictable focal periodicity and a precarious potential to cause large epidemics (Tsegaye et al., 2018).

Regarding entomological indices, cross-sectional sampling was undertaken and thus it was not possible to assess the relative importance of each breeding site over time or with respect to the rainy season; nor was it logistically feasible to extend mosquito collections to the forest, to improve our understanding of potential local sylvatic YFV transmission. It was also not feasible to retrieve historical climatic data during the YFV outbreak period, which could have helped to identify environmental risk factors for this outbreak. Due to the relatively low numbers of individual mosquitoes collected, it was not possible to identify the vector species responsible for YFV transmission nor successfully detect any arboviruses within sampled mosquitoes. Data from migrant workers and nomadic populations may not have been captured within this study, partly due to the time of day in which the questionnaires were completed, and therefore a follow up survey should be conducted, also including those working for the Omo Kuraz Sugar Factory in Salamago. All interviews were conducted by the HEWs in the language most comfortable to the interviewee. However, in each kebele we worked with different HEWs to ensure community acceptability, but this may have introduced interview bias when completing the questionnaire, translating and interpreting answers. Due to small numbers of participants from each kebele, data were pooled for analysis; however, this may have concealed variations in KAP level by location, therefore multivariate analysis was performed to account for this. Finally, self-reporting of vaccination status also introduced an unascertainable amount of recall bias, but unfortunately, vaccination cards were not provided during the mass campaign.

## Conclusions

Despite YFV re-emerging in recent years, little research has been conducted across endemic countries, particularly in Ethiopia. As evidenced by the ongoing YFV outbreak in 2018, the SOZ area in South-West Ethiopia still remains at risk for YFV transmission. With the high density of *Aedes* mosquitoes breeding close to human habitats, and relatively low community knowledge levels of YF prevention methods, study findings highlight the importance of providing information to these at-risk communities to encourage more effective and sustainable vector control practices. Further research needs to be conducted in the surrounding areas, to identify the major vector species of YFV, to understand the local sylvatic YFV transmission in forest areas and to further define the local distribution of *A. simpsoni* complex mosquitoes. Follow-up vaccination campaigns should be considered to target remaining pockets of potentially unvaccinated populations, in parallel to introducing the vaccination into the country’s national childhood immunization regimen for endemic areas of the country. Future training of HEWs and health professionals is necessary to ensure sustained high knowledge of both professionals and the community. Overall, study results emphasize the need to strengthen local and national disease surveillance and in-country laboratory capacity to ensure more rapid detection and response to future outbreaks.

## Supplemental Information

10.7717/peerj.6466/supp-1 Supplemental Information 1 ITS2 sequence files.

10.7717/peerj.6466/supp-2 Supplemental Information 2 Arbovirus screening assays including PCR primer/probes sequences and cycling conditions.

10.7717/peerj.6466/supp-3 Supplemental Information 3 Household Questionnaire: English Version.

10.7717/peerj.6466/supp-4 Supplemental Information 4 Knowledge of YFV signs, symptoms and transmission modes among study respondents in South Omo Zone, Ethiopia, 2017 (*n* = 180).

10.7717/peerj.6466/supp-5 Supplemental Information 5 Attitudes of study respondents towards YFV in South Omo Zone, Ethiopia, 2017 (*n* = 180).

10.7717/peerj.6466/supp-6 Supplemental Information 6 Practices for YFV prevention and mosquito control among study respondents in South Omo Zone, Ethiopia, 2017 (*n* = 180).

## Additional Information and Declarations

### DNA Deposition

The following information was supplied regarding the deposition of DNA sequences: The ITS2 sequences described here are accessible via GenBank, accession numbers MH277621–MH277635.

### Data Availability

The following information was supplied regarding data availability: Messenger, Louisa (2018): KAP_Data copy.xlsx. figshare. Dataset. DOI 10.6084/m9.figshare.7335815.v1.
