# Supplementary material for: A community-level investigation following a yellow fever virus outbreak in South Omo Zone, South-West Ethiopia
Source: PeerJ. 2019 Feb 20;7:e6466. doi: 10.7717/peerj.6466 (PMC6387579; doi:10.7717/peerj.6466)
Supplement: Supplemental Information 2 [file peerj-07-6466-s002.docx]

**Table S1.** Arbovirus screening assays including PCR primer/probes sequences and cycling conditions.

| **Pathogen** | **Primer and probe sequences** | **Cycling conditions** | **Reference** |
| --- | --- | --- | --- |
| DENV | 5′-TTGAGTAAACYRTGCTGCCTGTAGCTC-3  5′-GAGACAGCAGGATCTCTGGTCTYTC-3′ | 95°C for 10 min  50 cycles: 95°C for 10 sec, 60°C for 10 sec, 72°C for 10 sec | [1] |
| ZIKV | 5’- CCGCTGCCCAACACAAG -3’  5’- CCACTAACGTTCTTTTGCAGACAT -3’  5’-FAM-AGCCTACCTTGACAAGCAGTCAGACACTCAA-TAMRA3’ | 95°C for 10 min  45 cycles: 95°C for 10 sec, 52°C for 30 sec | [2] |
| CHIKV | 5’- CTCATA CCGCATCCGCATCAG-3’  5’- ACATTGGCCCCACAAT GAATTTG-3’ | 95°C for 10 min  40 cycles: 95°C for 10 sec, 56°C for 10 sec, 72°C for 15 sec | [3] |
| WNV | 5’-CCTGTGTGAGCTGACAAACTTAGT-3’  5’-GCGTTTTAGCATATTGACAGCC-3’  5’-6FAM-CCTGGTTTCTTAGACATCGAGATCT-[TAMRA]-3’ | 95°C for 15 min  45 cycles: 94°C for 15 sec, 60°C for 60 sec | [4] |
| YFV | 5’- AATCGAGTTGCTAGGCAATAAACAC-3’  5’- TCCCTGAGCTTTACGACCAGA-3’ | 95°C for 10 min  40 cycles: 95°C for 10 sec, 58°C for 10 sec, 72°C for 10 sec | [5] |
| RVFV | 5’-CTAGCCGTTTCACAAACTGGG-3’  5’-GACTGARGAYTCTGAATTGCACC-3’ | 95°C for 10 min  45 cycles: 95°C for 10 sec, 60°C for 10 sec, 72°C for 20 sec | [6] |

1. Lai YL, Chung YK, Tan HC, Yap HF, Yap G, Ooi EE, et al. Cost-effective real-time reverse transcriptase PCR (RT-PCR) to screen for Dengue virus followed by rapid single-tube multiplex RT-PCR for serotyping of the virus. J Clin Microbiol. 2007/01/12. 2007;45: 935–941. doi:10.1128/JCM.01258-06

2. Lanciotti RS, Kosoy OL, Laven JJ, Velez JO, Lambert AJ, Johnson AJ, et al. Genetic and serologic properties of Zika virus associated with an epidemic, Yap State, Micronesia, 2007. Emerg Infect Dis. 2008;14: 1232–1239. doi:10.3201/eid1408.080287

3. Ali UH, Vasan SS, Thayan R, Angamuthu C, Lim LH, Sekaran SD. Development and evaluation of a one-step SYBR-Green I-based real-time RT-PCR assay for the detection and quantification of Chikungunya virus in human, monkey and mosquito samples. Trop Biomed. 2010;27: 611–623.

4. Linke S, Ellerbrok H, Niedrig M, Nitsche A, Pauli G. Detection of West Nile virus lineages 1 and 2 by real-time PCR. J Virol Methods. 2007;146: 355–358. doi:10.1016/j.jviromet.2007.05.021

5. Dash PK, Boutonnier A, Prina E, Sharma S, Reiter P. Development of a SYBR green I based RT-PCR assay for yellow fever virus: application in assessment of YFV infection in Aedes aegypti. Virol J. 2012/01/24. 2012;9: 27. doi:10.1186/1743-422X-9-27

6. Maquart M, Temmam S, Héraud J-M, Leparc-Goffart I, Cêtre-Sossah C, Dellagi K, et al. Development of real-time RT-PCR for the detection of low concentrations of Rift Valley fever virus. J Virol Methods. 2014;195: 92–99. doi:10.1016/j.jviromet.2013.10.001
