# Supplementary material for: A community-level investigation following a yellow fever virus outbreak in South Omo Zone, South-West Ethiopia
Source: PeerJ. 2019 Feb 20;7:e6466. doi: 10.7717/peerj.6466 (PMC6387579; doi:10.7717/peerj.6466)
Supplement: Supplemental Information 3 [file peerj-07-6466-s003.docx]

**Supplementary File S2: Household Questionnaire: English Version**

| **Details of the household** | | | |
| --- | --- | --- | --- |
| Date |  | Initials of fieldworker: |  |
| Kebele & Cluster No: |  | Head of House (HoH) name: |  |
| Household No: |  |  |  |
| GPS Coordinates |  | Interviewee name, if not HoH: |  |

| **1. Socio-demographic characteristics of study participant (circle or tick as appropriate)** | | | |
| --- | --- | --- | --- |
| Q1. Age Group | <20 | Q2. Sex | Male |
|  | 20-24 |  | Female |
|  | 25-29 | Q3. Marital Status | Married |
|  | 30-34 |  | Single |
|  | 35-39 |  | Divorced |
|  | >40 |  | Widowed |
| Q4. Education | Illiterate/ None | Q5. Occupation | Unemployed / Housewife |
|  | Primary |  | Farmer |
|  | Secondary |  | Shopkeeper / Vendor |
|  | Higher Education/University |  | Forest-worker |
| Q6. How many people living in your household? (including participant) | 4 |  | Fisherman |
|  | 5 |  | Teacher |
|  | 6 |  | Student |
|  | Other: |  | Other: |

| **2. Knowledge of yellow fever (YF) symptoms, signs and transmission modes (circle as appropriate)** | | | | | |
| --- | --- | --- | --- | --- | --- |
| Q7. Have you heard of yellow fever (YF)? | Yes | | No | | |
| **2.1 Signs and symptoms** | | | | | |
| Q8. Is fever a symptom of YF? | Yes | No | | | Don’t know |
| Q9. Is headache a symptom of YF? | Yes | No | | | Don’t know |
| Q10. Is jaundice a symptom of yellow fever? | Yes | No | | | Don’t know |
| Q11. Is muscle pain a symptom of YF? | Yes | No | | | Don’t know |
| Q12. Is rash a symptom of YF? | Yes | No | | | Don’t know |
| Q13. Is bloody vomiting a symptom of YF? | Yes | No | | | Don’t know |
| Q14. Are there any other symptoms that have not been listed? (Please specify) |  | | | | |
| **2.2 Transmission** | | | | | |
| Q15. Do mosquitoes transmit YF? | Yes | No | | | Don’t know |
| Q16. Are they the same mosquitoes that transmit malaria? | Yes | No | | | Don’t know |
| Q17. Does ordinary person to person contact transmit YF? | Yes | No | | | Don’t know |
| Q18. Is YF transmitted through food and water? | Yes | No | | | Don’t know |
| Q19. When are the YF mosquitoes most likely to feed/bite? | Night | Day | | Both | Don’t Know |
| Q20. Do the YF mosquitoes breed in standing water? | Yes | No | | | Don’t know |
| Q21. Can mosquitoes breed inside the home? | Yes | No | | | Don’t know |
| Q22. Does removal or covering of standing water prevent mosquito breeding? | Yes | No | | | Don’t know |
| Q23. Can pouring chemicals into standing water kill mosquito larvae? | Yes | No | | | Don’t know |

| **3. Attitudes towards yellow fever** | | |
| --- | --- | --- |
| Q24. Is yellow fever a serious illness? | Yes | No |
| Q24b. If yes, why: | | |
| Q25. Are you at risk of YF where you live? | Yes | No |
| Q25b. If yes, why: | | |
| Q26. What disease are you most fearful of getting? | Malaria | Typhoid |
|  | Yellow Fever | Bacterial infection |
|  | Pneumonia | Regular fever |
|  | Other (please specify): | |
| Q27. Is controlling the breeding sites of mosquitoes a good strategy to prevent YF? | Yes | No |
| Q28. Is vaccination a good strategy to prevent YF? | Yes | No |
| Q29. Do you think communities should actively participate in controlling the mosquitoes of YF? | Yes | No |
| Q30. Do you think it’s the responsibility of the administrative & health office to control and prevent YF? | Yes | No |

| **4. Preventative practices against yellow fever** | | | | | |
| --- | --- | --- | --- | --- | --- |
| **4.1 Preventing mosquito-man contact** | | | | | |
| Q31. Do you do anything to reduce mosquitoes? (If yes, please answer the questions below. If no, then proceed to “*Eliminating breeding sites”*) | | Yes | | No | |
| Q31a. Has the government come to spray insecticide to reduce mosquitoes? | | Yes | | No | |
| Q31b. Do you prevent standing water around the house to reduce mosquitoes? | | Yes | | No | |
| Q31c. Do you use insecticide treated nets to protect against mosquitoes in the home? | | Yes | | No | |
| Q31d. Do you use of smoke to drive mosquitoes away? | | Yes | | No | |
| Q31e. Do you cover your body with clothes to protect against mosquitoes? | | Yes | | No | |
| **4.2 Eliminating Breeding Sites** | | | | | |
| Q32. Do you cover water containers in the home? | Yes | | | No | |
| Q33. How often do you clean water filed containers and ditches around the house? | Everyday | | Once a week | Once a month | Never |
| Q34. Do you turn containers upside down to avoid water collection? | Yes | | | No | |
| Q35. Do you do anything else to protect against mosquitoes? (please specify) | | | | | |

| **(5) Sources of information regarding yellow fever** | | | | |
| --- | --- | --- | --- | --- |
| Q36. Where do you normally get your information about yellow fever? | TV | Radio | Health Extension Workers | Religious Leader |
|  | Loudspeaker | Brochure | Friends | Internet |
|  | Other: | | | |

| **(6) Yellow Fever Case Finding** | | |
| --- | --- | --- |
| Q37. Have you ever had malaria? | Yes | No |
| Q38. Have you ever had YF? | Yes | No |
| Q38b. If yes, when: | | |
| Q38c. If yes, what treatment did you receive and from whom (health centre / hospital / traditional healer / other) | | |
| Q39. Do you know anyone who has had YF? | Yes | No |
| Q39b. If yes, who and when: | | |
| Q39c. If yes, what is their profession? | | |

| **(7) Vaccination Coverage Estimation** | | |
| --- | --- | --- |
| Q40. Have you ever been vaccinated against YF? | Yes | No |
| Q40b. If yes, do you remember where or when you had it? (Was it at your health post?) | | |
| Q41. Has anyone else in your household been vaccinated against YF? | Yes | No |
| Q41b. If yes, who and when? (Please name ALL members of your household who have been vaccinated, their ages and any other details e.g. where, when) | | |

| **(8) Other epidemiological risk factors** | | |
| --- | --- | --- |
| Q42. Do you work in Mago National Park / other forest areas? | Yes | No |
| Q43. Have you had any contact with monkeys? | Yes | No |
| Q44. Is there presence of false banana around the home? | Yes | No |
| Q45. Do you store water in open-containers inside or outside the home? | Yes | No |
| Q46. Have you (and your family) recently migrated to this area? | Yes | No |
| Q46b. If yes, from where, when and why? | | |

Do you have any other questions or comments you would like to make?

____________________________________________________________________________________________________________________________________________________________________________________________________________________________________________________________________________________________________
