# Supplementary material for: A community-level investigation following a yellow fever virus outbreak in South Omo Zone, South-West Ethiopia
Source: PeerJ. 2019 Feb 20;7:e6466. doi: 10.7717/peerj.6466 (PMC6387579; doi:10.7717/peerj.6466)
Supplement: Supplemental Information 4 [file peerj-07-6466-s004.docx]

**Table S3:** Knowledge of YFV signs, symptoms and transmission modes among study respondents in South Omo Zone, Ethiopia, 2017 (n=180).

|  |  |  |
| --- | --- | --- |
| KNOWLEDGE | **Frequency** | **Percentage %** |
| Have you heard of yellow fever? |  |  |
| Yes | 158 | 87.8 |
| No | 22 | 12.2 |
| SIGNS AND SYMPTOMS |  |  |
| Is fever a symptom of YFV? |  |  |
| Yes | 148 | 82.2 |
| No | 8 | 4.5 |
| Don’t know | 2 | 1.1 |
| No answer | 22 | 12.2 |
| Is headache a symptom of YFV? |  |  |
| Yes | 148 | 82.2 |
| No | 8 | 4.5 |
| Don’t know | 2 | 1.1 |
| No answer | 22 | 12.2 |
| Is jaundice a symptom of YFV? |  |  |
| Yes | 111 | 61.7 |
| No | 37 | 20.5 |
| Don’t know | 10 | 5.6 |
| No answer | 22 | 12.2 |
| Is muscle pain a symptom of YFV? |  |  |
| Yes | 134 | 74.5 |
| No | 20 | 11.1 |
| Don’t know | 4 | 2.2 |
| No answer | 22 | 12.2 |
| Is rash a symptom of YFV? |  |  |
| Yes | 97 | 53.9 |
| No | 50 | 27.8 |
| Don’t know | 11 | 6.1 |
| No answer | 22 | 12.2 |
| Is bloody vomiting a symptom of YFV? |  |  |
| Yes | 101 | 56.1 |
| No | 29 | 16.1 |
| Don’t know | 28 | 15.6 |
| No answer | 22 | 12.2 |

| TRANSMISSION | Frequency | Percentage % |
| --- | --- | --- |
| Do mosquitoes transmit YFV? |  |  |
| Yes | 149 | 82.8 |
| No | 9 | 5.0 |
| Don’t know | 3 | 1.7 |
| No answer | 19 | 10.5 |
| Are they the same mosquitoes that transmit malaria? |  |  |
| Yes | 81 | 45.0 |
| No | 54 | 30.0 |
| Don’t know | 26 | 14.4 |
| No answer | 19 | 10.6 |
| Does ordinary person to person contact transmit YFV? |  |  |
| Yes | 103 | 57.2 |
| No | 46 | 25.6 |
| Don’t know | 12 | 6.6 |
| No answer | 19 | 10.6 |
| Is YFV transmitted through food and water? |  |  |
| Yes | 38 | 21.1 |
| No | 113 | 62.8 |
| Don’t know | 10 | 5.5 |
| No answer | 19 | 10.6 |
| When are the YFV mosquitoes most likely to bite? |  |  |
| Night | 106 | 58.9 |
| Day | 16 | 8.9 |
| Both | 39 | 21.6 |
| Don’t know | 19 | 10.6 |
| Do the YFV mosquitoes breed in standing water? |  |  |
| Yes | 151 | 83.9 |
| No | 2 | 1.1 |
| Don’t know | 8 | 4.4 |
| No answer | 19 | 10.6 |
| Can mosquitoes breed inside the home? |  |  |
| Yes | 84 | 46.7 |
| No | 64 | 35.6 |
| Don’t know | 13 | 7.1 |
| No answer | 19 | 10.6 |
| Does removal or covering of standing water prevent mosquito breeding? |  |  |
| Yes | 138 | 76.7 |
| No | 10 | 5.5 |
| Don’t know | 13 | 7.2 |
| No answer | 19 | 10.6 |
| Can pouring chemical into standing water kill mosquito larvae? |  |  |
| Yes | 145 | 80.6 |
| No | 6 | 3.3 |
| Don’t know | 10 | 5.5 |
| No answer | 19 | 10.6 |
