# Supplementary material for: A community-level investigation following a yellow fever virus outbreak in South Omo Zone, South-West Ethiopia
Source: PeerJ. 2019 Feb 20;7:e6466. doi: 10.7717/peerj.6466 (PMC6387579; doi:10.7717/peerj.6466)
Supplement: Supplemental Information 5 [file peerj-07-6466-s005.docx]

**Table S4:** Attitudes of study respondents towards YFV in South Omo Zone, Ethiopia, 2017 (n=180).

| ATTITUDES | Frequency | Percentage % |
| --- | --- | --- |
| Is yellow fever a serious illness? |  |  |
| Yes | 147 | 81.7 |
| No | 16 | 8.9 |
| No answer | 17 | 9.4 |
| Is controlling the breeding sites of mosquitoes a good strategy to prevent YFV? |  |  |
| Yes | 157 | 87.2 |
| No | 7 | 3.9 |
| No answer | 16 | 8.9 |
| Is vaccination a good strategy to prevent YFV? |  |  |
| Yes | 136 | 75.6 |
| No | 28 | 15.6 |
| No answer | 16 | 8.8 |
| Do you think communities should actively participate in controlling the mosquitoes of YFV? |  |  |
| Yes | 156 | 86.7 |
| No | 8 | 4.4 |
| No answer | 16 | 8.8 |
| Do you think its responsibility of the health office to control and prevent YFV? |  |  |
| Yes | 101 | 56.1 |
| No | 63 | 35.0 |
| No answer | 16 | 8.9 |
