# Supplementary material for: A community-level investigation following a yellow fever virus outbreak in South Omo Zone, South-West Ethiopia
Source: PeerJ. 2019 Feb 20;7:e6466. doi: 10.7717/peerj.6466 (PMC6387579; doi:10.7717/peerj.6466)
Supplement: Supplemental Information 6 [file peerj-07-6466-s006.docx]

**Table S5:** Practices for YFV prevention and mosquito control among study respondents in South Omo Zone, Ethiopia, 2017 (n=180).

| PREVENTING MOSQUITO-MAN CONTACT | Frequency | Percentage % |
| --- | --- | --- |
| Do you do anything to reduce mosquitoes? |  |  |
| Yes | 166 | 92.2 |
| No | 14 | 7.8 |
| Has the government come to spray insecticide to reduce mosquitoes? |  |  |
| Yes | 145 | 80.6 |
| No | 35 | 19.4 |
| Do you prevent standing water around the house to reduce mosquitoes? |  |  |
| Yes | 161 | 89.4 |
| No | 19 | 10.6 |
| Do you use insecticide treated nets to protect against mosquitoes in the home? |  |  |
| Yes | 154 | 85.6 |
| No | 26 | 14.4 |
| Do you use smoke to drive mosquitoes away? |  |  |
| Yes | 156 | 86.7 |
| No | 24 | 13.3 |
| Do you cover your body with clothes to prevent against mosquito bites? |  |  |
| Yes | 167 | 92.8 |
| No | 13 | 7.2 |
| ELIMINATING BREEDING SITES |  |  |
| Do you cover water containers in the home? |  |  |
| Yes | 162 | 90.0 |
| No | 18 | 10.0 |
| How often do you clean water filled containers and ditches around the house? |  |  |
| Everyday | 67 | 37.3 |
| Once a week | 93 | 51.7 |
| Once a month | 9 | 5.0 |
| Never | 7 | 3.9 |
| No answer | 4 | 2.1 |
| Do you turn containers upside down to avoid water collection? |  |  |
| Yes | 152 | 84.4 |
| No | 27 | 15.0 |
| No answer | 1 | 0.6 |
